# Plasma exchange with albumin replacement for Alzheimer's disease treatment induced changes in serum and cerebrospinal fluid inflammatory mediator levels

**DOI:** 10.1002/acn3.52235

**Published:** 2024-10-30

**Authors:** Ricardo Gonzalo, Carla Minguet, Ana María Ortiz, María Isabel Bravo, Oscar L. López, Mercè Boada, Agustín Ruiz, Montserrat Costa

**Affiliations:** ^1^ Grifols Scientific Innovation Office Avinguda de la Generalitat 152–158 Sant Cugat del Vallès 08174 Barcelona Spain; ^2^ Departments of Neurology and Psychiatry University of Pittsburgh School of Medicine 811 Kaufmann Medical Building, 3471 Fifth Avenue Pittsburgh 15213 Pennsylvania USA; ^3^ ACE Alzheimer Centre Barcelona ‐ Universitat Internacional de Catalunya Gran Via de Carles III, 85 BIS Barcelona 08028 Spain; ^4^ Centro de Investigación Biomédica en Red de Enfermedades Neurodegenerativas (CIBERNED) Instituto de Salud Carlos III Calle de Melchor Fernández Almagro 3, Fuencarral‐El Pardo Madrid 28029 Spain

## Abstract

**Objective:**

There is extensive literature indicating that inflammatory pathways are affected in Alzheimer's disease (AD). We examined whether plasma exchange with albumin replacement (PE‐Alb) can impact the inflammatory status of AD patients and alter the relationship between inflammatory mediators and cognitive measures.

**Methods:**

Serum and cerebrospinal fluid (CSF) samples from 142 AD patients participating in the AMBAR trial (14‐month schedule of PE‐Alb treatment vs. placebo [sham PE‐Alb]) were analyzed for changes from baseline for 19 inflammatory mediators (6 inflammatory cytokines, 9 chemokines, and 4 vascular injury indicators) at representative time points across the AMBAR study (lasting effects) as well as in pre‐ versus post‐PE‐Alb procedure (acute effects). Association between mediator changes and clinical outcomes reported in the AMBAR study (cognitive, functional, behavioral function, and global change tests) was assessed.

**Results:**

PE‐Alb significantly reduced IFN‐γ, eotaxin, MIP‐1α and ICAM‐1 levels in serum, and eotaxin‐3 and MIP‐1β levels in CSF, at various time points during treatment (*p* < 0.05; false discovery rate‐corrected). Vascular injury indicators were the mediators mostly affected by post‐ versus pre‐PE‐Alb level reduction. Increased serum MIP‐1α levels were associated with worsening in ADAS‐Cog, CDR‐sb, and ADCS‐CGIC scores in the placebo group, but not in the PE‐Alb‐treated group.

**Interpretation:**

Peripheral intervention could affect AD by reducing inflammatory mediators in both peripheral and central compartments. Changes in MIP‐1α due to PE‐Alb were associated with changes in clinical outcomes.

## Introduction

The AMBAR (Alzheimer Management By Albumin Replacement) study (EudraCT# 2011‐001598‐25; ClinicalTrials.gov ID: NCT01561053) used a therapeutic approach for Alzheimer's disease (AD) based on plasma exchange (PE) with albumin replacement (PE‐Alb).^1^ Clinical efficacy and safety results of the AMBAR study demonstrated that PE‐Alb approach was feasible and safe in mild‐to‐moderate AD patients.^2^ It stabilized, slowed the decline, or even improved AD cognitive and/or functional symptoms,^3^, ^4^ and it stabilized brain perfusion.^5^ The initial rationale behind the PE‐Alb‐based strategy was that routine removal of AD patient's plasma and, consequently, elimination of peripheral amyloid‐beta peptide (Aβ) species would favor the efflux of Aβ from brain to plasma,^6^ where Aβ circulates bound to albumin.^7^, ^8^ Moreover, the use of therapeutic albumin as the replacement fluid would promote further sequestration of Aβ and exert antioxidant and immunomodulatory effects by modulating intracellular (endosomal) pathways involved in the production of cytokines.^9^

PE is used to treat multiple systemic and CNS diseases that involve alteration of the immune system.^10^, ^11^ Therefore, it is expected that PE will remove other pathogenic substances originating from multiple dysregulated pathways in AD, including circulating prooxidant products, proinflammatory cytokines, and soluble adhesion molecules.^12^ Preliminary research studies around AMBAR support a multi‐mechanistic effect of PE‐Alb on patients' serum and CSF biological profiles including changes in proteins,^13^ lipids,^14^ and metabolites.^15^

Concerning AD pathogenesis, there is growing evidence of a role of not only Aβ plaques and neurofibrillary tangles, but also of a chronic inflammatory response in the AD brain. This response has been linked to the release of inflammatory cytokines, chemokines, and other toxic substances by activated microglia cells.^16^, ^17^ Moreover, a state of systemic inflammation has been proposed to start or accelerate neurodegenerative processes that eventually result in cognitive decline and AD.^18^ Therefore, AD pathogenesis may not be restricted to the neuronal compartment but may also involve peripheral inflammatory mechanisms.

The AMBAR trial provides a unique opportunity to examine the profile and changes over time for inflammatory mediators in AD patients treated with PE‐Alb compared to placebo. The availability of serum samples before and after each PE‐Alb treatment allowed to determine the acute effect of PE‐Alb on inflammatory mediators. Moreover, serum and CSF inflammatory mediator measurements at baseline, throughout and at the end of the trial allowed us to determine the lasting effects of the treatment. This study will further to our understanding of the positive clinical effects shown in the AMBAR trial and will contribute to deciphering the multi‐mechanistic basis responsible for these effects.

## Methods

### Study design

This study was performed in serum and CSF samples obtained from patients participating in the AMBAR study – 322 individuals diagnosed with mild‐to‐moderate AD (Mini‐Mental State Examination [MMSE]^19^ score from 18 to 26).^4^ Full demographic and clinical characteristics of these patients and the inclusion and exclusion criteria have been published elsewhere.^1^, ^4^ These patients underwent a 14‐month schedule of PE‐Alb treatment for AD.^4^ Institutional review boards (IRBs) or ethics committees from the AMBAR participating sites and health authorities approved the protocol, the patient information sheets, and the informed consent form, in agreement with the Declaration of Helsinki as well as the standards of Good Clinical Practice. The patient and a close relative or legal representative read the patient information sheet, agreed to participation in the trial, and then signed the informed consent form.

In the present analysis, the levels of select inflammatory mediators (proinflammatory cytokines, chemokines, and vascular injury indicators) were measured.^20^ Levels before and after PE‐Alb and changes from baseline at several representative time points across the AMBAR study were assessed. Finally, associations were assessed between inflammatory mediator levels and the clinical endpoints reported in the AMBAR study.^4^

### Plasma exchange treatment program

**Figure 1:**
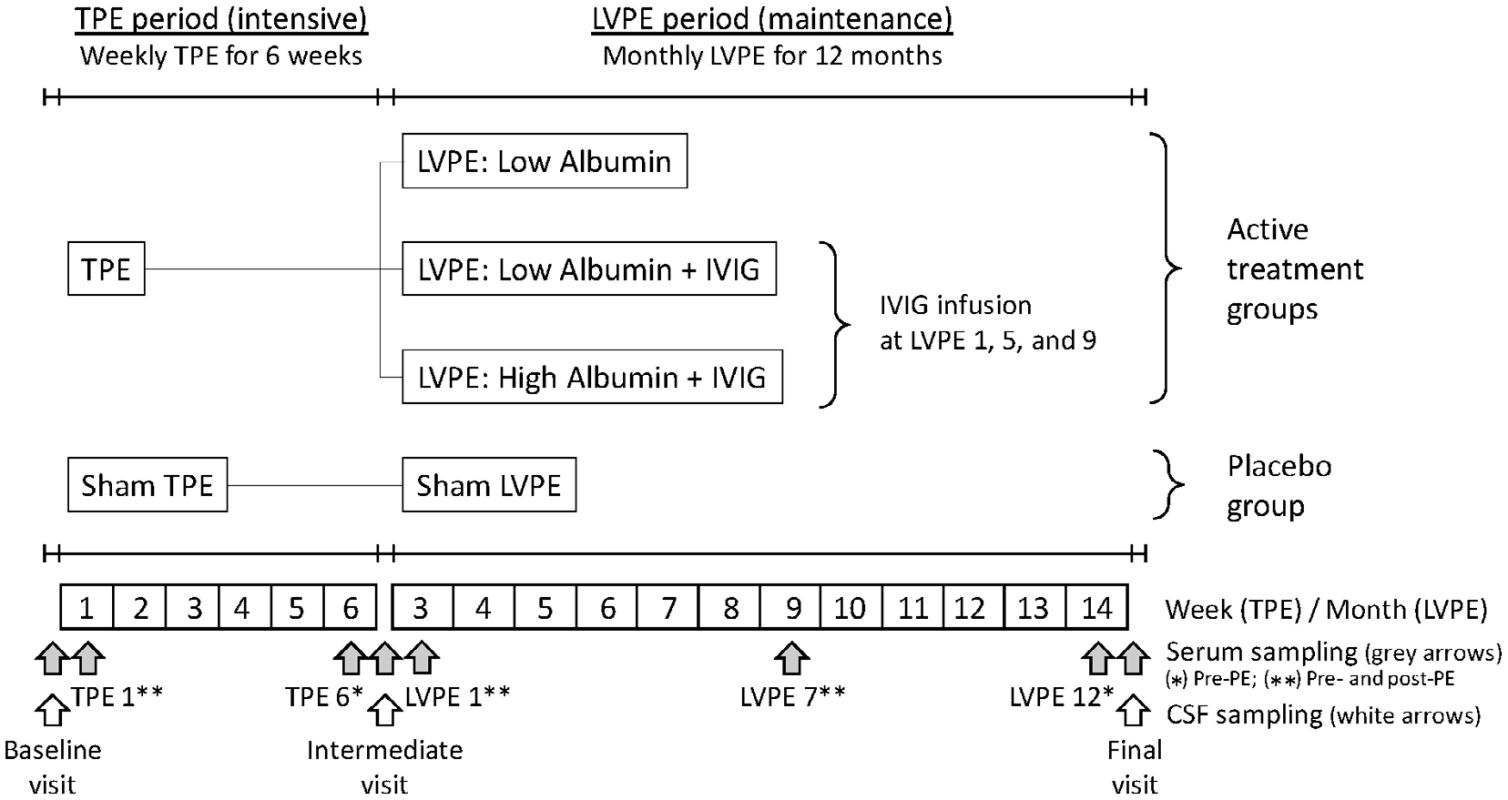
Outline of treatment periods, plasma exchange sessions, visits, and distribution of treatment groups in the AMBAR trial. Visits with serum (shaded arrows) and cerebrospinal fluid (blank arrows) samples available for the present study are indicated. IVIG, intravenous immunoglobulin; LVPE, low‐volume plasma exchange; TPE, therapeutic plasma exchange.

In the AMBAR trial, patients were randomized to one of three PE‐Alb treatments or to placebo (sham PE‐Alb) in a 1:1:1:1 fashion. The intervention regimen included a baseline visit, an initial 6‐week intensive period with one session of conventional therapeutic PE‐Alb (TPE; processing 1 plasma volume [2500–3000 mL]) per week (6 TPE sessions total) for all groups. This was followed by an intermediate visit where no PE‐Alb treatment was performed. Then a 12‐month maintenance period was performed with one session of low‐volume PE‐Alb (LVPE; removal of approximately one‐third plasma volume [650 to 880 mL]) per month (12 LVPE sessions in total) with three PE‐Alb treatment modalities: low‐dose albumin (*n* = 78), low‐dose albumin plus IVIG (*n* = 86), and high‐dose albumin plus IVIG (*n* = 78), plus the placebo group (*n* = 80). In the two treatment arms receiving IVIG, this was given instead of albumin in one of every four LVPE sessions (LVPE 1, LVPE 5, and LVPE 9). A final follow‐up visit at Month 14 closed the study. Treatment periods and treatment groups are summarized in Figure 1. Full details of the apheresis interventions in the AMBAR trial are available elsewhere.^1^, ^4^

### Patients and sampling

The AMBAR study recruited AD patients from 2012 to 2017. Serum and CSF samples were collected and stored at −80°C. However, at that temperature, cytokines degrade to 75% or less of baseline values after 4 years of storage [18]. As samples of this study were analyzed in 2020, only those patients whose samples were collected during 2016 and 2017 were considered (*N* = 142 patients). Table S1 shows the demographic and clinical features of the patients included in this study. Eligibility of the patients is shown in Figure S1.

For inflammatory mediator analysis, serum samples were collected at eight visits across the PE‐Alb treatment period. In three of the visits, samples were taken before and after the PE‐Alb session. In total, there were 11 time points for serum sampling: baseline (Month 0): TPE 1 (Month 0.2: pre‐ and post‐TPE); TPE 6 (Month 1.5: pre‐TPE); intermediate visit (Month 2); LVPE 1 (Month 2.2: pre‐ and post‐LVPE); LVPE 7 (Month 9: pre‐ and post‐LVPE); LVPE 12 (Month 13.5: pre‐LVPE); and final visit (Month 14). CSF samples were taken at three time points: baseline, intermediate, and final visits. Overall, that represented a total of 1674 samples for analysis: 1312 serum (eight visits) and 362 CSF (three visits). PE‐Alb treatment schedule and sampling points are summarized in Figure 1.

### Inflammatory mediator assays

Three electrochemiluminescence MSD V‐PLEX Panel kits (Meso Scale Diagnostic LLC; Rockville MD; USA) were used for analysis of inflammatory mediators in serum and CSF. These included a proinflammatory cytokine panel (Proinflammatory Panel 1 Human kit: interferon [IFN]‐γ; interleukin [IL]‐1β; IL‐2; IL‐4; IL‐6; IL‐8; IL‐10; IL‐12p70; IL‐13; and tumor necrosis factor [TNF]‐α), a chemokine panel (Chemokine Panel 1 Human kit: eotaxin; eotaxin‐3; IFN‐γ‐inducer protein [IP]‐10; monocyte chemoattractant protein [MCP]‐1; MCP‐4; macrophage‐derived chemokine [MDC]; macrophage inflammatory protein [MIP]‐1α; MIP‐1β; and thymus‐ and activation‐regulated chemokine [TARC]), and a vascular injury panel (Vascular injury Panel 2 Human kit: serum amyloid A [SAA]; C‐reactive protein [CRP]; vascular cell adhesion protein [VCAM]‐1; and intercellular adhesion molecule [ICAM]‐1), for a total of 23 inflammatory mediators.

As a quality control criterion, inflammatory mediators were considered evaluable only if they were detectable (i.e., excluding values below lower limit of quantification and missing values) in more than 30% (ad‐hoc value) of the samples in all the time points evaluated.

### Clinical outcomes

Outcome endpoints (clinical tests score) obtained in the primary study^4^ that were of interest for this analysis were as follows: Alzheimer's Disease Assessment Scale‐Cognitive Subscale (ADAS‐Cog) that evaluates cognitive and behavioral function; Alzheimer's Disease Cooperative Study – Activities of Daily Living (ADCS‐ADL) that evaluates functional ability; Clinical Dementia Rating Sum of Boxes (CDR‐sb) that evaluates global change, and Alzheimer's Disease Cooperative Study‐Clinical Global Impression of Change (ADCS‐CGIC) that evaluates cognitive, functional, and behavioral function. In the evaluated patients, the possible association between the levels of serum and CSF inflammatory mediators and the clinical at baseline, intermediate, LVPE 7, and final visits, was investigated.

### Data analysis and statistics

The baseline distribution of the analyzed inflammatory mediators' levels in the placebo and the PE‐Alb‐treated groups were compared by means of a Wilcoxon test.

Evaluation of treatment effects was performed by considering the lasting effects (i.e., effects of PE‐Alb treatment over time) and acute effects (i.e., effects of PE‐Alb treatment associated to each single PE session: pre‐ and post‐PE‐Alb).

The lasting effects were analyzed in pooled PE‐Alb treated patients compared to placebo as the change from baseline levels through a mixed model for repeated measures (MMRM). For this analysis, only samples pre‐PE‐Alb were considered. Following variables were used as fixed effects factors: “Visit” defined units as months, “Treatment group” with placebo group as reference, and “Treatment group × Visit” interaction, with adjustment for age, AD severity (baseline MMSE score), and baseline inflammatory mediator levels, and “patient” variable was included as a repeated factor in the model defined as follows:

$$\mathrm{IM}=\begin{array}{l} \alpha +\beta_{1}\;\text{Visit}+\beta_{2}\;\text{Treatment group}\\ +\;\beta_{3}\;\text{Treatment}\_\text{group}\times \text{Visit}+\beta_{4}\;\mathrm{Age}+\beta_{5}\;{\text{MMSE}}_{0}\\ +\;\beta_{6}\;{\mathrm{IM}}_{0}+\left(1|\text{Patient}\right). \end{array}$$

where, IM, inflammatory mediator level; IM_0_, inflammatory mediator level at baseline; Treatment_group, placebo or PE‐Alb‐treated; Visit, Month 0, 0.2, 1.5, 2, 2.2, 9, 13.5, and 14 for serum samples and Month 0, 2, and 14 for CSF samples; MMSE_0_, baseline MMSE score. Estimate for the Treatment_group × Visit interaction (*β*_3_) was used to evaluate the longitudinal effect of treatment on the inflammatory mediators.

In addition, heatmap charts were created from the ratio between groups (pooled PE‐Alb‐treated versus placebo) of the least squares mean (LSM) previously calculated. All measures were log2 normalized and standardized prior to analyses so that β‐coefficients of the interaction Treatment_group × Visit can be interpreted as standardized effect size.

Acute effects were assessed in samples collected immediately before and after PE‐Alb in those sessions where post‐PE‐Alb samples were available: TPE 1, LVPE 1, and LVPE 7. Samples for acute effect analysis were pooled in TPE 1 and LVPE 7 visits, but not for LVPE 1, as in this PE‐Alb session two of the treated arms received IVIG (See Fig. 1). Effect size between samples collected before and after the PE‐Alb session, was calculated as the rank biserial correlation for nonparametric test.

The possible relationship between changes in the levels of serum and CSF inflammatory mediators across the study and the clinical endpoints was investigated through a repeated measures correlation analysis (“rmcorr” R package) as well as through a parsimonious MMRM defined as follows:

$${\mathrm{CFB}}_{\text{Clinical endpoint}}=\begin{array}{l} \alpha +\beta_{1}\;\text{Visit}\\ +\;\beta_{2}\;\text{Treatment group}+\beta_{3}\;\mathrm{IM}\\ +\;\beta_{4}\cdot \mathrm{IM}\times \text{Treatment}\_\text{group}\\ \times \text{Visit}+\left(1|\text{Patient}\right). \end{array}$$

where, CFB, change from baseline value; IM, inflammatory mediator level; Treatment_group, PE‐Alb‐treated or placebo; Visit, Month 0, 2, 9, or 14 for serum samples and Month 0, 2, and 14 for CSF samples.

An extended MMRM that included all the variables used in the MMRM for the lasting effects analysis previously described was further applied to those inflammatory mediators which were statistically significant in the parsimonious model to evaluate the possible influence of the other variables in the relationship. The extended MMRM was defined as follows:

$${\mathrm{CFB}}_{\text{Cinical endpoint}}=\begin{array}{l} \alpha +\beta_{1}\;\text{Visit}\\ +\;\beta_{2}\;\text{Treatment group}+\beta_{3}\;\mathrm{IM}\\ +\;\beta_{4}\cdot \mathrm{IM}\times \text{Treatment}\_\text{group}\\ \times \text{Visit}+\beta_{5}\;{\text{MMSE}}_{0}+\beta_{6}\;\mathrm{Age}\\ +\;\beta_{7}\;\text{Clinical}\_{\text{Endpoint}}_{0}\\ +\;\left(1|\text{Patient}\right). \end{array}$$

where, MMSE_0_, baseline MMSE score; Clinical_Endpoint_0_, clinical endpoint value at baseline. Estimate for the IM × Treatment_group × Visit triple interaction (*β*_4_) was used to evaluate the relationship between changes in the levels of serum and CSF inflammatory mediators and the clinical endpoints across the study in parsimonious and extended model.

To confirm that the statistical differences found in the correlation between inflammatory mediators and the clinical outcome were not derived or influenced by other measured variables, a mediation analysis was performed. Study demographic and laboratory variables were added to the previous parsimonious model to test its influence on the statistical significance and on the *β*_4_ estimate (IM × Treatment_group × Visit interaction). Prediction plots from the parsimonious MMRM were generated using “effects” R package to illustrate the estimated effects of predictor variables MIP‐1α (macrophage inflammatory protein 1‐alpha) and treatment on the response variable, the clinical outcome endpoints assessed as change from baseline.

When indicated, *p* values were adjusted by Benjamini–Hochberg procedure to account for multiple comparisons and decrease the false discovery rate. A *p* value below 0.05 was used as statistical significance threshold. All statistical analyses were performed with R version 4.1 (https://cran.r‐project.org/).

## Results

### Patients' clinical characteristics

Demographics and baseline characteristics of the cohort of patients from the AMBAR trial whose samples were included in this study are shown in Table S1.

**Figure 2:**
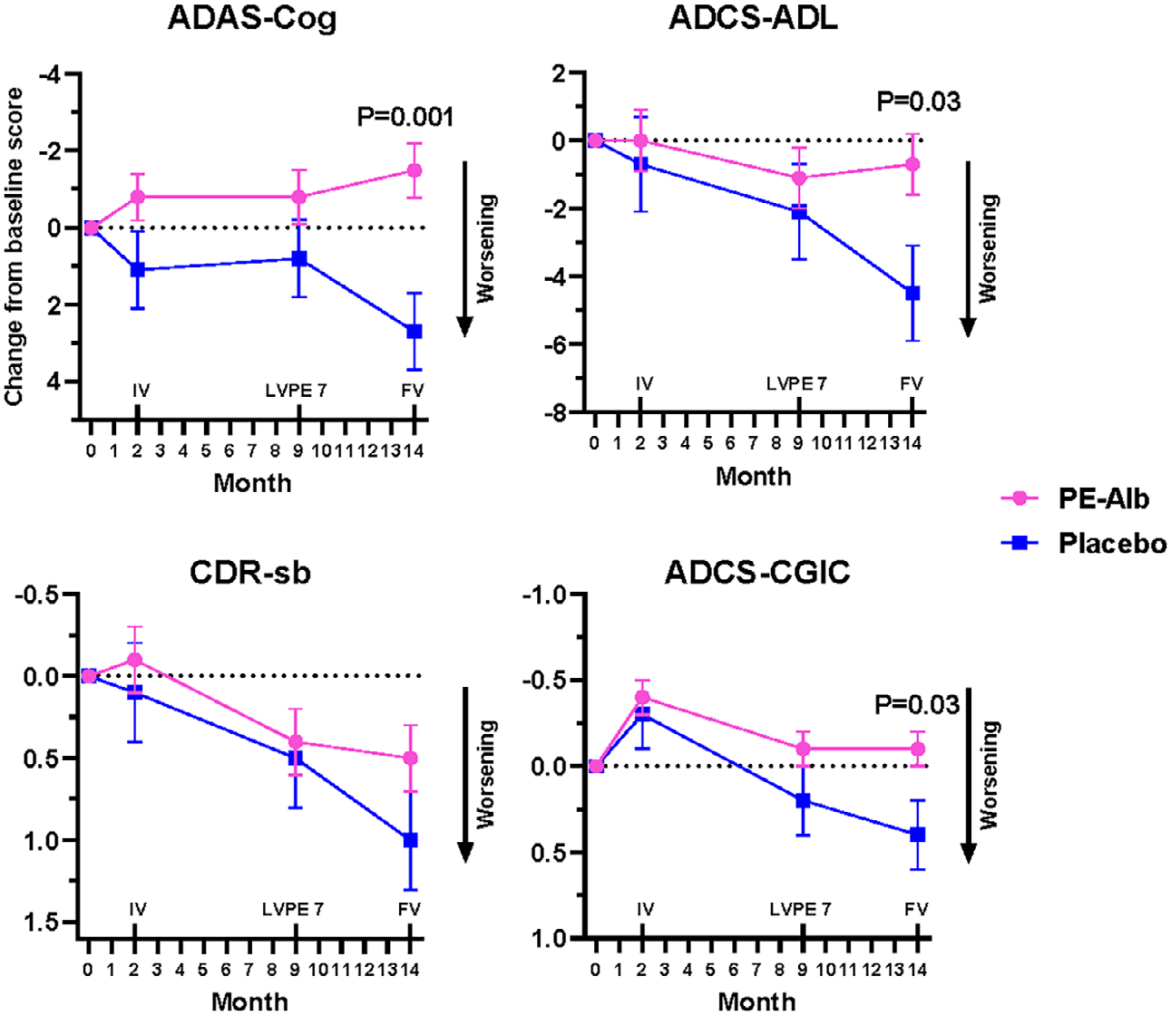
Change from baseline in clinical outcome tests score (ADAS‐Cog, ADCS‐ADL, CDR‐sb, and ADCS‐CGIC) in the patient cohort of this study. FV, final visit (end of LVPE period); IV, intermediate visit (end of therapeutic plasma exchange period); LVPE 7, low‐volume plasma exchange 7; PE‐Alb, plasma exchange with albumin replacement.

Change from baseline values of ADAS‐Cog, ADCS‐ADL, CDR‐sb, and ADCS‐CGIC tests score at the scheduled visits in this study cohort are summarized in Figure 2. In all tests, the placebo group scored consistently toward worsening, whereas the PE‐Alb‐treated group scores showed less worsening or stabilization, with statistically significance in three of the tests.

### Evaluable mediators

In the panel of ten proinflammatory cytokines, six in serum and two in CSF were deemed evaluable. In the panel of nine chemokines, all were evaluable in serum and five in CSF. All four inflammatory mediators in the vascular injury panel were evaluable for both matrices. Overall, out of the 23 inflammatory mediators, 19 were evaluable in serum and 11 in CSF. Details of mediators deemed evaluable and non‐evaluable according to the percentage of assessable samples (i.e., excluding those with an undetectable value and those unavailable due to missing values) are shown in Table S2 (serum) and Table S3 (CSF).

### Baseline levels: Treated versus placebo

At baseline, there were no statistically significant differences between PE‐Alb‐treated and placebo samples of serum and CSF for any of the assessable inflammatory mediators. All inflammatory mediator levels are available in Figure S2 (serum) and Figure S3 (CSF).

### Lasting effect

**Figure 3:**
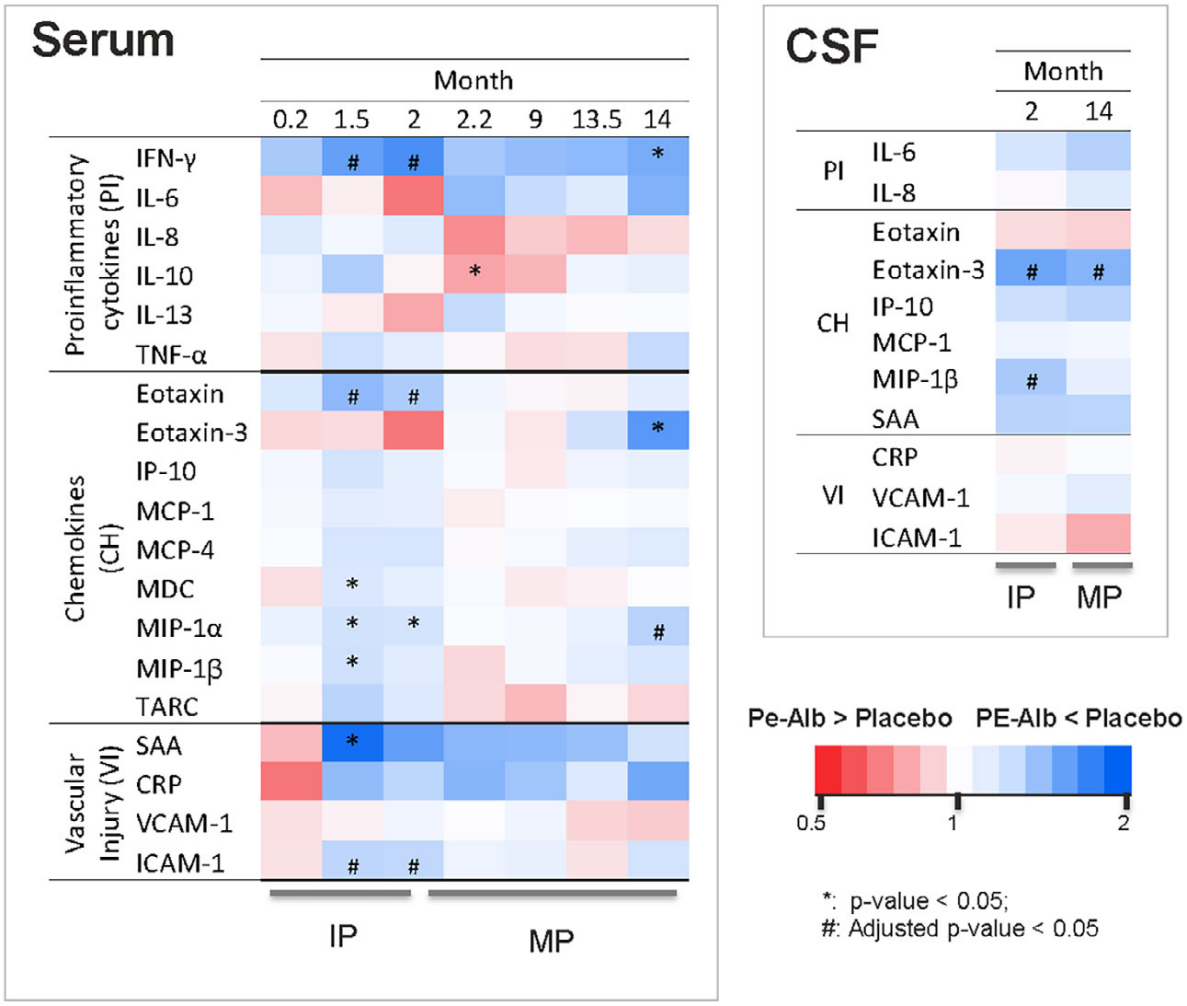
Heatmap representation of least squares mean ratio between the group of patients treated with plasma exchange with albumin replacement (PE‐Alb; *n* = 54–102) and placebo group (*n* = 16–36). Gradient in red means decreasing ratio <1 (favors placebo) whereas gradient in blue means increasing ratio >1 (favors PE‐Alb treatment). See Figure 1 for details of treatment periods, visits, and treatment patient groups in the AMBAR trial. CSF, cerebrospinal fluid; IP, intensive period (therapeutic plasma exchange); MP, maintenance period (low‐volume plasma exchange).

The heatmap generated from the LSM ratio between PE‐Alb‐treated versus placebo groups for both serum and CSF is shown in Figure 3 (source LSM plots are available in Figs. S4 and S5), with gradient in blue indicating a PE‐Alb treatment favoring effect and a gradient in red indicating the opposite.

In serum, blue color (PE‐Alb treatment favoring effect) was predominant at the conclusion of the intensive period (month 2) for 15 inflammatory mediators, and remained so at the end of study (Month 14) for 13 inflammatory mediators. Statistically significant differences from baseline favoring PE‐Alb treatment (*p* < 0.05; raw *p* value) for at least one time point (Month 0.2, 1.5, 2, 2.2, 9, 13.5, and 14) were observed with nine inflammatory mediators (IFN‐γ; IL‐10; eotaxin; eotaxin‐3; MDC, MIP‐1α; MIP‐1β; SAA; and ICAM‐1). IL‐10 (an anti‐inflammatory cytokine) was the only mediator for which significance favored placebo. After BH adjustment, differences remained significant for IFN‐γ (Months 1.5 and 2), eotaxin (Month 1.5), MIP‐1α (Month 14), and ICAM‐1 (Months 1.5 and 2).

In CSF, most inflammatory mediators (8 of 11) showed an effect favoring PE‐Alb treatment at the end of intensive period and the end of study. Two (eotaxin‐3 and MIP‐1β) showed statistically significant differences (*p* < 0.05; BH adjustment) (Fig. 3).

### Acute effects

Levels of all analyzed serum inflammatory mediators remained unchanged after sham PE‐Alb in the placebo samples from the three sessions assessed for acute effects (TPE 1, LVPE 1, and LVPE 7).

**Figure 4:**
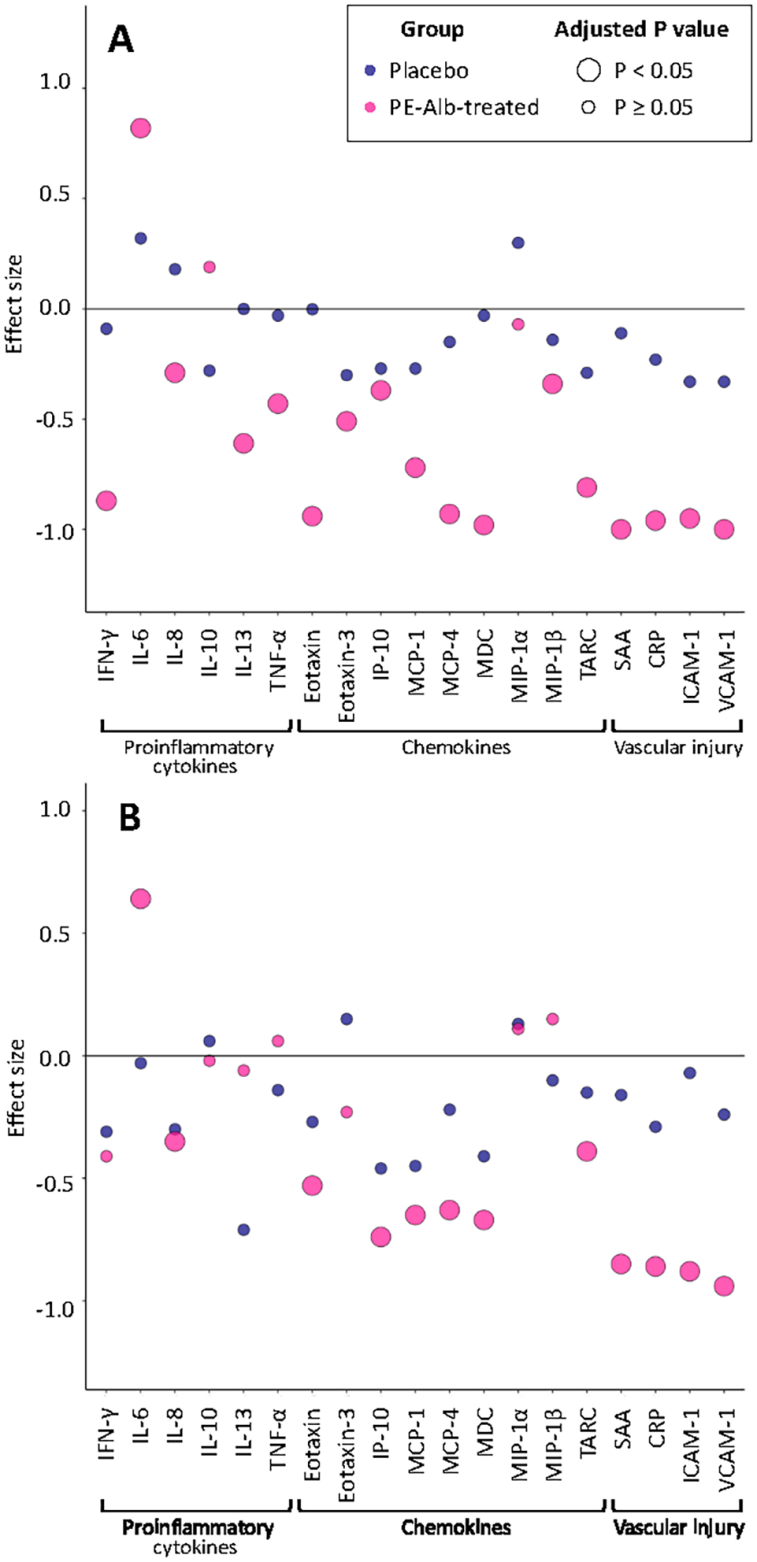
Plots of the changes in inflammatory mediator levels (effect size in PE‐Alb‐treated with respect to placebo) before and after PE‐Alb (acute effects). (A) TPE 1 (therapeutic plasma exchange 1; Month 0.2); (B) LVPE 7 (low‐volume plasma exchange 7; Month 9).

Conversely, 16 of 19 and 11 of 19, respectively, inflammatory mediator levels decreased significantly in serum samples from the PE‐Alb‐treated group (*p* < 0.05, BH adjustment) after TPE 1 and LVPE 7. Interestingly, IL‐6 levels were significantly increased. These results are summarized in Tables S3 and S4 and in plots for TPE 1 and LVPE 7 in Figure 4.

In LVPE 1, the two PE‐Alb‐treated arms that received IVIG (see Fig. 1), showed significant increases in most serum inflammatory mediators in the proinflammatory cytokine and chemokine panels. Conversely, mediators in the vascular injury panel decreased significantly in all three PE‐Alb‐treated arms. Details are provided in Figure S6.

### Association with clinical outcome studies

MMRM analysis showed that serum MIP‐1α was the only one of the 19 serum inflammatory mediators assessed that showed a significant inverse association (*p* < 0.05; raw *p* value) with change from baseline values for three of the four clinical outcomes (ADAS‐Cog, CDR‐sb, and ADCS‐CGIC). Six other inflammatory mediators showed a statistically significant association with at least one of the tests. The results indicating significant association were consistent in both the parsimonious and extended MMRM models. Beta for the triple interaction term (*β*_4_) and *p* values for all inflammatory mediators and clinical outcome tests are shown in Table 1 (parsimonious model) and Table S5 of the Supporting Information (extended model).

**Table 1:** Longitudinal association of serum inflammatory mediator levels and treatment with clinical outcome tests score (ADAS‐Cog, ADCS‐ADL, CDR‐sb, and ADCS‐CGIC) using a mixed model for repeated measures (parsimonious model). Beta for the triple interaction term (*β*_4_) and *p* values are indicated.

Mediator	ADAS‐Cog		ADCS‐ADL		CDR‐sb		ADCS‐CGIC	
	*β*_4_	*p*	*β*_4_	*p*	*β*_4_	*p*	*β*_4_	*p*
Proinflammatory cytokines								
IFN‐γ	0.27	0.324	−0.09	0.713	0.21	**0.010**	0.01	0.798
IL‐6	0.06	0.588	−0.05	0.658	0.02	0.618	0.01	0.604
IL‐8	−0.08	0.445	0.46	**0.001**	−0.01	0.807	0	0.832
IL‐10	−0.09	0.716	0.43	0.059	0.12	0.127	0.02	0.601
IL‐13	0.16	0.370	−0.06	0.744	0.03	0.567	0.02	0.510
TNF‐α	0.11	0.446	−0.05	0.782	0.03	0.459	0.03	0.238
Chemokines								
Eotaxin	0.12	0.457	−0.06	0.767	0.06	0.266	−0.01	0.874
Eotaxin‐3	−0.13	0.471	−0.33	0.166	−0.33	0.162	−0.01	0.819
IP‐10	0	0.999	0.07	0.796	0.03	0.688	−0.01	0.896
MCP‐1	0.13	0.471	−0.19	0.415	0.12	**0.036**	0.01	0.837
MCP‐4	0.17	0.347	−0.18	0.437	0.12	**0.031**	0	0.912
MDC	−0.25	0.364	0.68	0.061	−0.14	0.136	−0.05	0.415
MIP‐1α	−0.73	**0.021**	0.52	0.236	−0.24	**0.021**	−0.16	**0.011**
MIP‐1β	0.11	0.587	−0.17	0.524	0.01	0.845	0.03	0.404
TARC	0.09	0.490	−0.08	0.627	0.01	0.810	0.01	0.792
Vascular injury indicators								
SAA	−0.06	0.512	0.16	0.222	0.01	0.721	0.02	0.332
CRP	−0.01	0.867	−0.14	0.225	0.02	0.485	0.03	**0.045**
ICAM‐1	0.37	0.234	−1.78	**<0.001**	0.15	0.127	0.10	0.085
VCAM‐1	−0.06	0.843	−0.33	0.408	0.11	0.263	0.05	0.327

In bold: *p* values with statistical significance (*p* < 0.05).

Repeated measures correlation results for serum showed that placebo samples had a positive correlation (r values ranging from 0.22 to 0.33) between MIP‐1α levels and ADAS‐Cog, CDR‐sb (both statistically significant), and ADCS‐CGIC (borderline significance). Conversely, this correlation was not observed in PE‐Alb‐treated samples. These results are summarized in Table 2.

**Table 2:** Repeated measures correlation of serum MIP‐1α levels with clinical outcome tests score (ADAS‐Cog, ADCS‐ADL, CDR‐sb, and ADCS‐CGIC) in patients treated with plasma exchange with albumin replacement (PE‐Alb) and placebo. Repeated measures correlation coefficient (*r*) and *p* values are indicated.

Treatment group	ADAS‐Cog		ADCS‐ADL		CDR‐sb		ADCS‐CGIC	
	*r*	*p*	*r*	*p*	*r*	*p*	*r*	*p*
PE‐Alb	−0.081	0.267	0.058	0.433	−0.052	0.476	−0.061	0.519
Placebo	0.328	**0.002**	−0.175	0.102	0.293	**0.006**	0.223	0.096

In bold: *p* values with statistical significance (*p* < 0.05).

In CSF, 6 of the 11 inflammatory mediators showed a statistically significant association with one clinical outcome. None showed association with more than one test. Similar to serum, significance results agreed in the parsimonious (Table 3) and extended MMRM (Table S6 of the Supporting Information) models.

**Table 3:** Longitudinal association of cerebrospinal fluid inflammatory mediator levels and treatment with clinical outcome tests score (ADAS‐Cog, ADCS‐ADL, CDR‐sb, and ADCS‐CGIC) with a mixed model for repeated measures (parsimonious model). Beta for the triple interaction term (*β*_4_) and *p* values are indicated.

Mediator	ADAS‐Cog		ADCS‐ADL		CDR‐sb		ADCS‐CGIC	
	*β*_4_	*p*	*β*_4_	*p*	*β*_4_	*p*	*β*_4_	*p*
Proinflammatory cytokines								
IL‐6	−0.03	0.773	−0.06	0.674	−0.12	**0.002**	−0.04	0.103
IL‐8	−0.04	0.835	−0.23	0.415	0.17	**0.011**	0.06	0.136
IP‐10	−0.17	0.446	0.13	0.686	0.07	0.320	−0.04	0.283
Chemokines								
Eotaxin‐3	−0.33	0.354	0.95	0.065	0.02	0.852	0.13	0.128
Eotaxin	−0.49	0.110	0.48	0.195	−0.34	**<0.001**	−0.09	0.135
MCP‐1	0.17	0.496	0.12	0.733	−0.04	0.664	−0.04	0.454
MIP‐1β	0.01	0.957	−0.23	0.323	−0.17	**0.003**	−0.04	0.265
Vascular injury indicators								
SAA	−0.08	0.427	−0.34	**0.006**	−0.01	0.816	0.01	0.677
CRP	−0.11	**0.043**	−0.13	0.055	−0.03	0.151	−0.01	0.311
VCAM‐1	0.13	0.461	−0.13	0.610	0.04	0.531	0.04	0.289
ICAM‐1	0.11	0.451	−0.34	0.068	0.05	0.368	0.05	0.130

In bold: *p* values with statistical significance (*p* < 0.05).

**Figure 5:**
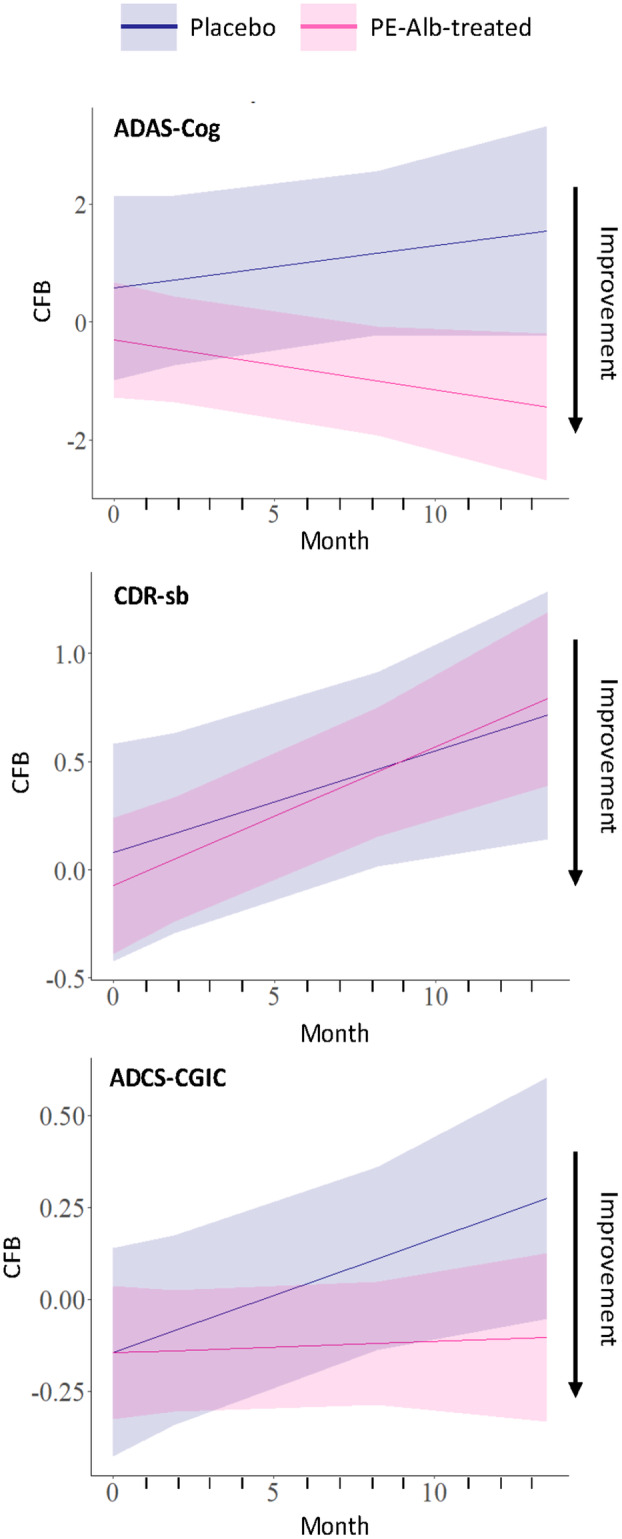
Predicted trajectories of clinical outcome (change from baseline [CFB] at the end of the study of clinical outcome tests score (ADAS‐Cog. CDR‐sb, and ADCS‐CGIG) according to levels of MIP‐1α in placebo and plasma exchange with albumin replacement (PE‐Alb)‐treated patients. Shaded areas represent the 95% confidence interval.

Predicted trajectories of clinical outcomes (change from baseline at the end of the study) based on levels of MIP‐1α are shown in Figure 5. ADAS‐Cog score showed a longitudinal improvement in PE‐Alb‐treated patients. CDR‐sb showed no clear differences between placebo and PE‐Alb‐treated patients. ADCS‐CGIC trajectories for placebo group showed worsening over time, while PE‐Alb‐treated patients remained stable.

To confirm that the longitudinal association found between MIP‐1α levels and clinical outcomes endpoints was not mediated by any other variable, a mediation analysis was conducted with demographic and biochemical variables. The statistical significance and estimates (*β*_4_) of the triple interaction term (IM × Treatment_group × Visit) of the parsimonious model were maintained regardless the variable assessed (see Fig. S7).

MIP‐1α could not be evaluated in CSF samples because it was assessable in only 13%–15% of the samples (see Table S3), that is, below the threshold of 30% (per protocol).

## Discussion

In the AMBAR trial, a novel PE‐Alb‐based strategy for the treatment of AD was developed. The basis of this treatment was a multi‐mechanistic approach that included the induction of Aβ efflux from brain to periphery and the removal of beta toxic species and other pathogenic substances resulting from oxidative stress and inflammatory events.^6^, ^9^, ^12^

This study showed for the first time that a peripheral intervention for the treatment of AD, such as PE‐Alb, can alter both peripheral and CNS inflammatory mediators with a subsequent positive effect on clinical outcomes. We found that (1) significant changes from baseline in inflammatory mediators mostly favored a decrease in inflammation in PE‐Alb‐treated patients in both serum and CSF; (2) There was a positive correlation between MIP‐1α levels and the decline of clinical cognition measures in the placebo arm, but not in the PE‐Alb‐treated arm; (3) the effect of changes in inflammatory mediators persisted long‐term during the study. These findings provide evidence that the positive clinical effects obtained in AMBAR are secondary to changes in multiple pathological pathways.

In this study, we characterized the profile and evolution of inflammatory mediators in serum and CSF samples from AMBAR patients across the trial. Furthermore, the correlation of the treatment effect on inflammatory mediators with clinical benefit was explored.

Only reliable inflammatory mediators (as defined in the protocol) were analyzed. First, we selected samples collected within the 4‐year period preceding analysis to avoid analysis of samples with significant cytokine degradation.^20^ Second, we considered an inflammatory mediator to be evaluable only if it was detected in at least the 30% of the samples. And third, baseline levels of the mediator were consistent between PE‐Alb‐treated patients and placebo. From 23 inflammatory mediators analyzed, this selection process resulting in 19 mediators evaluable in serum and 11 in CSF.

Acute effects showed that levels of most inflammatory mediators decreased immediately after the two PE‐Alb sessions assessed (TPE 1 and LVPE 7). Generally, a larger effect size was observed after TPE 1 than after LVPE 7, as reasonably expected given the different amounts of plasma exchanged (2500–3000 mL in TPE; 650 mL–880 mL in LVPE^1^). However, most mediators from proinflammatory cytokine and chemokine panels increased after LVPE 1. As altered expression of different inflammatory factors can either promote or counteract neurodegenerative processes,^12^ we cannot rule this out as the true beneficial effect of the treatment. Moreover, IVIG was administered in LVPE 1 in groups randomized to receive it, therefore a transient increase of proinflammatory cytokines could be induced by known adverse events related to IVIG infusion.^9^ Nevertheless, lasting effects benefited all PE‐Alb‐treated patients, as discussed in the next paragraph.

Lasting effects studies evidenced that PE‐Alb induced changes in patients' serum and CSF inflammatory profiles persisted across the study. Interestingly, IL‐10 was the only cytokine in serum which levels significantly increased with respect to placebo at least at one time point (although prior to *p* value correction). This would be in agreement with IL‐10 having potent anti‐inflammatory properties.^21^ In addition to IL‐10, all other inflammatory mediators tested showed significant decrease from baseline. This supports a reduction of the inflammation status in PE‐Alb‐treated patients versus placebo, although not fully homogeneous across all tested time points. This effect was particularly evidenced at Months 1.5 and 2 (end of intensive period and intermediate visit, respectively: significant decrease in INF‐γ, eotaxin, and ICAM‐1 in serum; eotaxin‐3 and MIP‐1β in CSF) and at Month 14 (the end of study: significant decrease in MIP‐1α in serum and eotaxin‐3 in CSF). Importantly, our results of lasting effects suggest that a peripheral intervention such as PE‐Alb can affect the levels of inflammatory mediators in the CSF.

In assessing the relevance of these inflammatory mediators in AD, the scientific literature provides controversial results. As reviewed by Brosseron et al, many of the mediators evaluated in our study have been reported to be upregulated, downregulated, or unchanged in AD.^22^ Recently, inflammatory plasma molecules have been reported by Koca et al. to be reduced in moderate‐stage AD patients (*n* = 25).^23^ Previously, a study by Choi et al.^24^ found eotaxin significantly elevated in AD patients (*n* = 11), and IFN‐γ and MIP‐1α being non‐significantly elevated, which is partially in agreement with our results. Nevertheless, much of these discrepancies may lie in the intrinsic instability of the cytokines or in the shortage of longitudinal data with large sample sizes. Our study along with the AMBAR trial contributes to filling this knowledge gap by analysis of inflammatory mediators over time, and also supports the multi‐mechanistic basis of the treatment. To support the finding of inflammatory mediator changes associated with PE‐Alb, their association with the AMBAR clinical outcome was assessed.

The association results suggested that serum MIP‐1α can be a candidate indicator of a PE‐Alb treatment efficacy in AD as performed in the AMBAR study. Changes in MIP‐1α levels in serum, as detected in repeated measures correlation and MMRM analyses, were significantly associated with changes in the three clinical outcome tests that include cognition (ADAS‐Cog, CDR‐sb, and ADCS‐CGIC) but not in the functionality test (ADCS‐ADL). MIP‐1α is a factor produced by macrophages and monocytes that is crucial for immune responses toward infection and inflammation.^25^ MIP‐1α is also produced by neurons and microglia.^26^ Interestingly, peripheral T cells of AD patients overexpress MIP‐1α to enhance its T cell migration from blood to brain.^27^ Further studies are warranted to confirm the relevance of serum MIP‐1α as a surrogate marker of PE‐Alb treatment efficacy for AD. Regrettably, MIP‐1α could not be evaluated in CSF samples. Therefore, the correlation of MIP1α among compartments remains to be investigated.

The fact that only samples collected during the last 2 years of the AMBAR study were viable limited the sample size for analysis and subanalyses. However, the patients with available samples (142 of 322, close to half of the recruited patients) were demographically and clinically comparable to the original AMBAR trial population.^4^ Another limitation is the reduced number of inflammatory biomarkers measured in the current study. Therefore, further research is needed to identify whether other possible inflammatory mediators are altered after PE‐Alb treatment in patients with AD.

In conclusion, results of this study indicate that the peripheral intervention performed on AD patients could induce both peripheral and central effects on inflammation by significantly reducing several proinflammatory cytokines, chemokines, and vascular injury indicators. Remarkably, changes in MIP‐1α levels correlated with the positive clinical outcomes. The findings of reduced inflammation in PE‐Alb‐treated AD patients should be confirmed in additional similar studies. Likewise, the potential of serum MIP‐1α as an indicator of PE‐Alb treatment efficacy for AD should be validated in another patient cohort.

## Author Contributions

RG processed the experimental data, performed the analysis, helped in drafting the manuscript and designed tables and figures. CM and AMO contributed to sample preparation, planned the experiments, and supervised the experimental work. MB and OLL were the Principal Investigators of the AMBAR study. MIB, MB, OLL, and AR. aided in interpreting the results and worked on the manuscript. MC conceived the original idea, designed the study and supervised the project. All authors discussed the results and contributed to the final manuscript.

## Conflict of Interest

RG, CM, MIB, and MC are full‐time employees of Grifols. AMO was a full‐time employee of Grifols when the study was performed. MB has received consultant fees from Araclon, Avid, Bayer, Elan, Grifols, Janssen/Pfizer, Lilly, Neuroptix, Nutricia, Roche, Sanofi, Biogen, and Servier; and received fees for lectures and funds for research from Araclon, Lilly, Grifols, Janssen, Novartis, Nutricia, Piramal, Pfizer‐Wyett, Roche, and Servier. OL has received consultant fees from Grifols and Lundbeck. AR has received been a consultant fees from Grifols, Prevail Therapeutics, and Landsteiner Genmed; reports grants/research funding from Abbvie, Janssen, Grifols, and Fundación Bancaria LaCaixa; and has stocks in Landsteiner Genmed.

## Supporting information


Appendix S1.

## Data Availability

Data reported in this manuscript are available within the article and/or its Supporting Information. Clinical data and/or results from the AMBAR study are available in the clinical trials registers (EudraCT#: 2011‐001598‐25; ClinicalTrials.gov ID: NCT01561053) and in the published papers (https://doi.org/10.1002/alz.12137; https://doi.org/10.1002/alz.12477; https://doi.org/10.1007/s00259‐022‐05915‐5). Additional data are available from the corresponding author upon reasonable request.
