# Supplementary material for: Plasma exchange with albumin replacement for Alzheimer's disease treatment induced changes in serum and cerebrospinal fluid inflammatory mediator levels
Source: Ann Clin Transl Neurol. 2024 Oct 30;11(12):3280–91. doi: 10.1002/acn3.52235 (PMC11651178; doi:10.1002/acn3.52235)
Supplement: Supplementary file 1 — Appendix S1. [file ACN3-11-3280-s001.docx]

# Supplementary material

**Table S1**. Demographic and clinical characteristics at baseline by treatment arm in the patient cohort of this study. Values of the original population of the AMBAR study are also shown for reference

| **Characteristic** | **Placebo** | **PE-Alb-treatment** | | | | **Total** |  |
| --- | --- | --- | --- | --- | --- | --- | --- |
|  |  | **Low Albumin** | **Low Albumin - IVIG** | **High Albumin + IVIG** | **All 3 treated groups** |  | **p-value^a^** |
| N | 37 | 36 | 34 | 35 | 105 | 142 |  |
| AMBAR | 80 | 78 | 86 | 78 | 242 | 322 |  |
| Age, years; mean (SD) | 67.0 (8.3) | 69.6 (7.1) | 68.7 (5.8) | 69.7 (8.1) | 69.1 (7.0) | 68.6 (7.4) | 0.562 |
| AMBAR | 68.4 (8.4) | 68.5 (7.5) | 69.5 (6.9) | 69.5 (7.9) | 69.2 (7.4) | 69.0 (7.7) |  |
| Sex: Male; n (%) | 15 (40.5) | 14 (38.9) | 19 (55.9) | 13 (37.1) | 46 43.8) | 61 (43.0) | 0.618 |
| AMBAR | 44 (55.0) | 35 (44.9) | 38 (44.2) | 31 (39.7) | 104 (43.0) | 148 (46.0) |  |
| Female; n (%) | 22 (59.5) | 22 (61.1) | 15 (44.1) | 22 (62.9) | 59 (56.2) | 81 (57.0) | 0.618 |
| AMBAR | 36 (45.0) | 43 (55.1) | 48 (55.8) | 47 (60.3) | 138 (57.0) | 174 (54.0) |  |
| MMSE score; mean (SD) | 22.0 (2.3) | 21.9 (2.4) | 22.5 (2.4) | 21.8 (2.5) | 22.1 (2.5) | 22.04 (2.4) | 0.087 |
| AMBAR | 21.7 (2.6) | 21.2 (2.4) | 22.1 (2.6) | 21.4 (2.6) | 21.7 (2.6) | 21.6 (2.6) |  |
| ApoE E4; n (%) | 36 (97.3) | 34 (94.4) | 34 (100) | 32 (91.4) | 102 (97.1) | 138 (97.2) | 0.599 |
| AMBAR | 71 (96.3) | 74 (94.9) | 77 (96.5) | 75 (96.2) | 226 (93.4) | 308 (95.7) |  |
| Carriers | 14 (37.8) | 24 (66.7) | 16 (47.1) | 11 (31.4) | 51 (48.6) | 65 (45.8) | 0.759 |
| AMBAR | 34 (44.2) | 47 (63.5) | 40 (48.2) | 33 (44.6) | 120 (51.9) | 154 (50.0) |  |
| Non-Carriers | 22 (59.5) | 10 (27.8) | 18 (52.9) | 23 (65.7) | 51 (48.6) | 73 (51.4) | 0.541 |
| AMBAR | 43 (55.8) | 27 (36.5) | 43 (51.8) | 41 (55.4) | 111 (49.1) | 154 (50.0) |  |
| CSF Aβ_42_, pg/ml;  median (IQR) | 598 (380-877) | 532 (452-666) | 623 (437-977) | 497 (403-875) | 544 (432-837) | 555 (432-857) | 0.103 |
| AMBAR | 551  (380-810) | 505 (431-700) | 502  (436-618) | 509  (399-730) | 505  (407-822) | 515 (426-737) |  |

IVIG: intravenous immunoglobulin; MMSE: Mini-Mental State Examination; CSF: cerebrospinal fluid; PE-Alb: plasma exchange with albumin replacement

^a^ p-values are referred to the comparison between total patient cohort of this study and the original AMBAR population.

**Table S2**. Evaluable inflammatory mediators in serum. A mediator was considered evaluable if the percentage of assessable samples (i.e., excluding those with an undetectable value and those unavailable due to missing value) was >30% in all visits

| **Mediator** | **% evaluable samples** | | | | | | | | | | | | | **Mediator evaluable in serum** |
| --- | --- | --- | --- | --- | --- | --- | --- | --- | --- | --- | --- | --- | --- | --- |
|  | **Baseline** | **TPE 1** | | | **TPE 6**  **Pre** | **Inter-mediate** | **LVPE 1** | | | **LVPE 7** | | **LVPE 12**  **Pre** | **Final** |  |
|  |  | **Pre** | **Post** | |  |  | **Pre** | | **Post** | **Pre** | **Post** |  |  |  |
| Proinflammatory cytokines | | | | | | | | | | | | | | |
| IFN-γ | 41 | 38 | | 46 | 41 | 34 | 41 | 55 | | 33 | 35 | 41 | 36 | Yes |
| IL-10 | 53 | 48 | | 53 | 39 | 51 | 56 | 63 | | 44 | 48 | 39 | 34 | Yes |
| IL-12p70 | 19 | 17 | | 31 | 13 | 13 | 15 | 31 | | 14 | 14 | 20 | 26 | No |
| IL-13 | 39 | 33 | | 43 | 33 | 41 | 97 | 61 | | 28* | 29* | 34 | 33 | Yes |
| IL-1β | 6 | 5 | | 26 | 5 | 5 | 4 | 25 | | 3 | 10 | 14 | 18 | No |
| IL-2 | 4 | 4 | | 20 | 1 | 0 | 3 | 14 | | 1 | 5 | 2 | 7 | No |
| IL-4 | 5 | 8 | | 28 | 6 | 5 | 6 | 25 | | 4 | 19 | 14 | 20 | No |
| IL-6 | 51 | 50 | | 73 | 47 | 45 | 40 | 75 | | 39 | 53 | 44 | 49 | Yes |
| IL-8 | 98 | 96 | | 97 | 96 | 95 | 96 | 97 | | 96 | 96 | 99 | 92 | Yes |
| TNF-α | 69 | 75 | | 77 | 80 | 73 | 74 | 79 | | 81 | 79 | 85 | 85 | Yes |
| Chemokines | | | | | | | | | | | | | | |
| Eotaxin | 99 | 98 | | 95 | 98 | 98 | 100 | 99 | | 99 | 95 | 99 | 98 | Yes |
| Eotaxin-3 | 53 | 49 | | 45 | 40 | 41 | 42 | 37 | | 36 | 42 | 26* | 28* | Yes |
| IP-10 | 81 | 80 | | 80 | 84 | 75 | 80 | 83 | | 68 | 67 | 79 | 56 | Yes |
| MCP-1 | 99 | 99 | | 98 | 100 | 100 | 100 | 97 | | 100 | 95 | 100 | 98 | Yes |
| MCP-4 | 99 | 95 | | 98 | 97 | 97 | 97 | 97 | | 96 | 95 | 99 | 96 | Yes |
| MDC | 99 | 99 | | 98 | 99 | 99 | 99 | 100 | | 100 | 98 | 100 | 98 | Yes |
| MIP-1α | 39 | 36 | | 39 | 39 | 39 | 49 | 69 | | 45 | 50 | 57 | 41 | Yes |
| MIP-1β | 99 | 96 | | 99 | 97 | 99 | 99 | 97 | | 98 | 98 | 99 | 98 | Yes |
| TARC | 91 | 89 | | 89 | 93 | 88 | 83 | 90 | | 81 | 81 | 89 | 88 | Yes |
| Vascular injury indicators | | | | | | | | | | | | | | |
| CRP | 88 | 92 | | 93 | 94 | 84 | 88 | 90 | | 91 | 89 | 70 | 84 | Yes |
| ICAM-1 | 91 | 88 | | 89 | 91 | 93 | 93 | 96 | | 92 | 92 | 72 | 81 | Yes |
| SAA | 89 | 86 | | 85 | 93 | 92 | 89 | 92 | | 91 | 94 | 99 | 96 | Yes |
| VCAM-1 | 94 | 92 | | 93 | 95 | 98 | 95 | 95 | | 91 | 92 | 83 | 91 | Yes |

* Exception to the rule of >30% cutoff to consider the mediator evaluable, because values were close to 30% and in 1 or 2 visits only

**Table S3**. Evaluable inflammatory mediators in CSF. A mediator was considered evaluable if the percentage of assessable samples (i.e., excluding those with an undetectable value and those unavailable due to missing value) was >30% in all visits

| **Mediator** | **% evaluable samples** | | | **Mediator evaluable in CSF** |
| --- | --- | --- | --- | --- |
|  | **Baseline** | **Inter-mediate** | **Final** |  |
| Proinflammatory cytokines | | | | |
| IFN-γ | 6 | 6 | 6 | No |
| IL-10 | 9 | 10 | 0 | No |
| IL-12p70 | 8 | 9 | 5 | No |
| IL-13 | 7 | 5 | 7 | No |
| IL-1β | 11 | 9 | 7 | No |
| IL-2 | 8 | 8 | 0 | No |
| IL-4 | 9 | 8 | 3 | No |
| IL-6 | 76 | 77 | 86 | Yes |
| IL-8 | 97 | 92 | 82 | Yes |
| TNF-α | 14 | 11 | 7 | No |
| Chemokines | | | | |
| Eotaxin | 58 | 48 | 46 | Yes |
| Eotaxin-3 | 73 | 42 | 31 | Yes |
| IP-10 | 76 | 77 | 91 | Yes |
| MCP-1 | 100 | 98 | 99 | Yes |
| MCP-4 | 9 | 10 | 1 | No |
| MDC | 13 | 15 | 13 | No |
| MIP-1α | 13 | 15 | 13 | No |
| MIP-1β | 80 | 81 | 81 | Yes |
| TARC | 14 | 15 | 13 | No |
| Vascular injury indicators | | | | |
| CRP | 90 | 89 | 59 | Yes |
| ICAM-1 | 89 | 86 | 56 | Yes |
| SAA | 35 | 40 | 23* | Yes |
| VCAM-1 | 94 | 92 | 93 | Yes |

* Exception to the rule of >30% cutoff to consider the mediator evaluable, because values were close to 30% and in one visit only

**Table S4**. Change in serum inflammatory mediator levels (effect size) in post- vs pre-TPE 1 (therapeutic plasma exchange 1; month 0.2) in patients treated with plasma exchange with albumin replacement and placebo

| **Mediator** | **Placebo** | | **PE-Alb-treated** | |
| --- | --- | --- | --- | --- |
|  | **P value (adjusted)** | **Effect size** | **P value (adjusted)** | **Effect size** |
| Proinflammatory cytokines | |  |  |  |
| IFN-γ | 1 | 0.09 | **<0.001** | 0.87 |
| IL-6 | 0.655 | -0.32 | **<0.001** | −0.82 |
| IL-8 | 0.655 | -0.18 | **0.021** | 0.29 |
| IL-10 | 0.655 | 0.28 | 0.304 | −0.19 |
| IL-13 | 1 | <0.001 | **0.032** | 0.61 |
| TNF-α | 1 | 0.03 | **0.003** | 0.43 |
| Chemokines |  |  |  |  |
| Eotaxin | 1 | 0.002 | **<0.001** | 0.94 |
| Eotaxin-3 | 0.655 | 0.30 | **0.004** | 0.51 |
| IP-10 | 0.655 | 0.27 | **0.009** | 0.37 |
| MCP-1 | 0.655 | 0.27 | **<0.001** | 0.72 |
| MCP-4 | 0.722 | 0.15 | **<0.001** | 0.93 |
| MDC | 1 | 0.03 | **<0.001** | 0.98 |
| MIP-1α | 0.655 | −0.30 | 0.682 | 0.07 |
| MIP-1β | 0.726 | 0.14 | **0.005** | 0.34 |
| TARC | 0.655 | 0.29 | **<0.001** | 0.81 |
| Vascular injury indicators | |  |  |  |
| SAA | 0.820 | 0.11 | **<0.001** | 1 |
| CRP | 0.655 | 0.23 | **<0.001** | 0.96 |
| ICAM-1 | 0.655 | 0.33 | **<0.001** | 0.95 |
| VCAM-1 | 0.655 | 0.33 | **<0.001** | 1 |

In bold: P values with statistical significance (P < 0.05); PE-Alb: plasma exchange with albumin replacement

**Table S5**. Change in serum inflammatory mediator levels (effect size) in post- vs pre-LVPE 7 (low-volume plasma exchange 7; month 9) in patients treated with plasma exchange with albumin replacement and placebo

| **Mediator** | **Placebo** | | **PE-Alb-treated** | |
| --- | --- | --- | --- | --- |
|  | **P value (adjusted)** | **Effect size** | **P value (adjusted)** | **Effect size** |
| Proinflammatory cytokines | |  |  |  |
| IFN-γ | 0.757 | 0.31 | 0.092 | 0.41 |
| IL-6 | 0.944 | 0.03 | **0.010** | −0.64 |
| IL-8 | 0.540 | 0.30 | **0.021** | 0.35 |
| IL-10 | 0.944 | −0.06 | 0.931 | 0.02 |
| IL-13 | 0.540 | 0.71 | 0.897 | 0.06 |
| TNF-α | 0.818 | 0.14 | 0.773 | −0.06 |
| Chemokines |  |  |  |  |
| Eotaxin | 0.540 | 0.27 | **<0.001** | 0.53 |
| Eotaxin-3 | 0.818 | −0.15 | 0.438 | 0.23 |
| IP-10 | 0.481 | 0.46 | **<0.001** | 0.74 |
| MCP-1 | 0.380 | 0.45 | **<0.001** | 0.65 |
| MCP-4 | 0.614 | 0.22 | **<0.001** | 0.63 |
| MDC | 0.380 | 0.41 | **<0.001** | 0.67 |
| MIP-1α | 0.818 | −0.13 | 0.616 | −0.11 |
| MIP-1β | 0.818 | 0.10 | 0.371 | −0.15 |
| TARC | 0.782 | 0.15 | **0.021** | 0.39 |
| Vascular injury indicators | |  |  |  |
| SAA | 0.757 | 0.16 | **<0.001** | 0.85 |
| CRP | 0.540 | 0.29 | **<0.001** | 0.86 |
| ICAM-1 | 0.834 | 0.07 | **<0.001** | 0.88 |
| VCAM-1 | 0.577 | 0.24 | **<0.001** | 0.94 |

In bold: P values with statistical significance (P < 0.05); PE-Alb: plasma exchange with albumin replacement

**Table S6**. Longitudinal association of serum inflammatory mediator levels and treatment with clinical outcome test scores (ADAS-Cog, ADCS-ADL, CDR-sb, ADCS-CGIC). MMRM extended model performed only with those mediators which association resulted statistically significant in the parsimonious model. Beta for the triple interaction term (β_4_) and P values are indicated

| **Mediator** | **ADAS-Cog** | | **ADCS-ADL** | | **CDR-sb** | | **ADCS-CGIC** | |
| --- | --- | --- | --- | --- | --- | --- | --- | --- |
|  | **β_4_** | **P** | **β_4_** | **P** | **β_4_** | **P** | **β_4_** | **P** |
| Proinflammatory cytokines | | | |  |  |  |  |  |
| IFN-γ | N/C | | N/C | | 0.22 | 0.010 | N/C | |
| IL-8 | N/C | | 0.44 | 0.001 | N/C | | N/C | |
| IL-10 | N/C | | 0.44 | 0.053 | N/C | | N/C | |
| Chemokines | | |  |  |  |  |  |  |
| MCP-1 | N/C | | N/C | | 0.12 | 0.034 | N/C | |
| MCP-4 | N/C | | N/C | | 0.12 | 0.029 | N/C | |
| MIP-1α | −0.69 | 0.028 | N/C | | −0.24 | 0.022 | −0.15 | 0.013 |
| Vascular injury indicators | | | |  |  |  |  |  |
| ICAM-1 | N/C | | −1.74 | <0.001 | N/C | | N/C | |

N/C: not calculated

**Table S7**. Longitudinal association of cerebrospinal fluid inflammatory mediators and treatment with clinical outcome tests score (ADAS-Cog, ADCS-ADL. CDR-sb, ADCS-CGIC). Mixed Model for Repeated Measures (extended model) performed only with those mediators which association resulted statistically significative in the parsimonious model. Beta for the triple interaction term (β_4_) and P values are indicated

| **Mediator** | **ADAS-Cog** | | **ADCS-ADL** | | **CDR-sb** | | **ADCS-CGIC** | |
| --- | --- | --- | --- | --- | --- | --- | --- | --- |
|  | **β_4_** | **P** | **β_4_** | **P** | **β_4_** | **P** | **β_4_** | **P** |
| Proinflammatory cytokines | | | |  |  |  |  |  |
| IL-6 | N/C | | N/C | | −0.12 | 0.002 | N/C | |
| IL-8 | N/C | | N/C | | 0.15 | 0.026 | N/C | |
| Chemokines | | |  |  |  |  |  |  |
| Eotaxin | N/C | | N/C | | −0.34 | <0.001 | N/C | |
| MIP-1β | N/C | | N/C | | −0.17 | 0.003 | N/C | |
| Vascular injury indicators | | | |  |  |  |  |  |
| SAA | N/C | | −0.35 | 0.004 | N/C | | N/C | |
| CRP | −0.11 | 0.045 | N/C | | N/C | | N/C | |

N/C: not calculated

**Fig S1. Flowchart of patients through the study.**

**
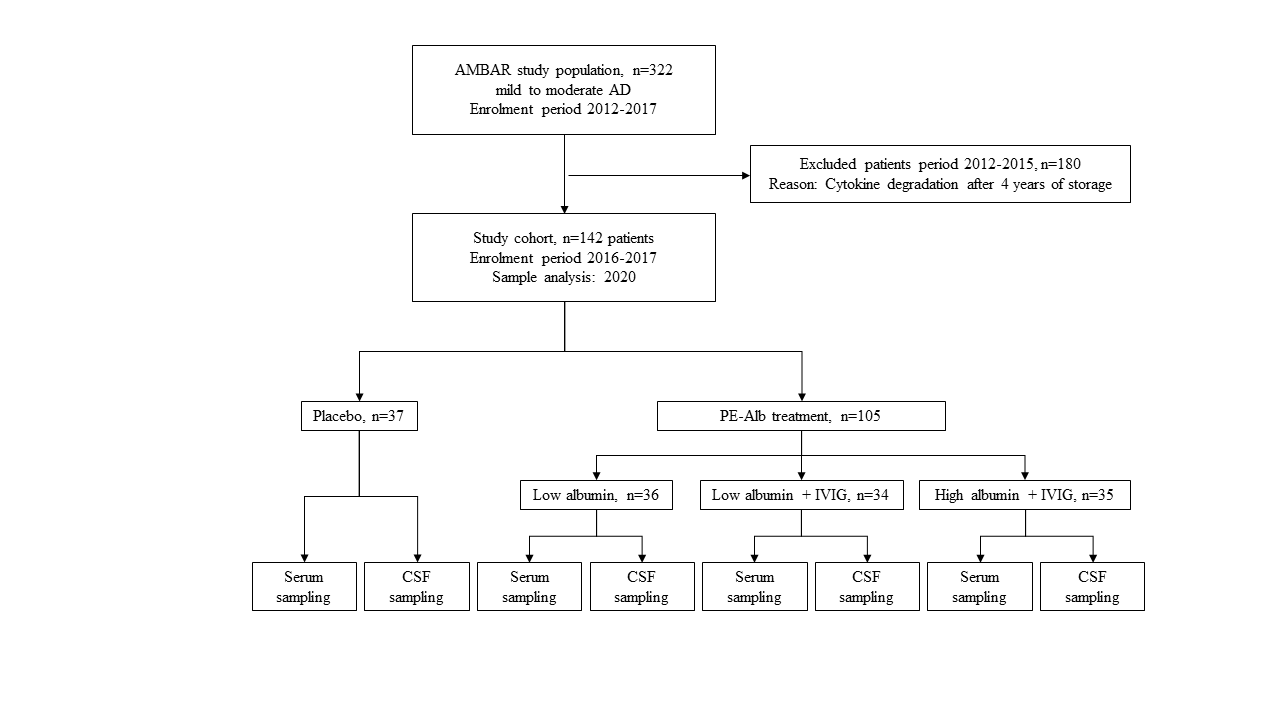
**


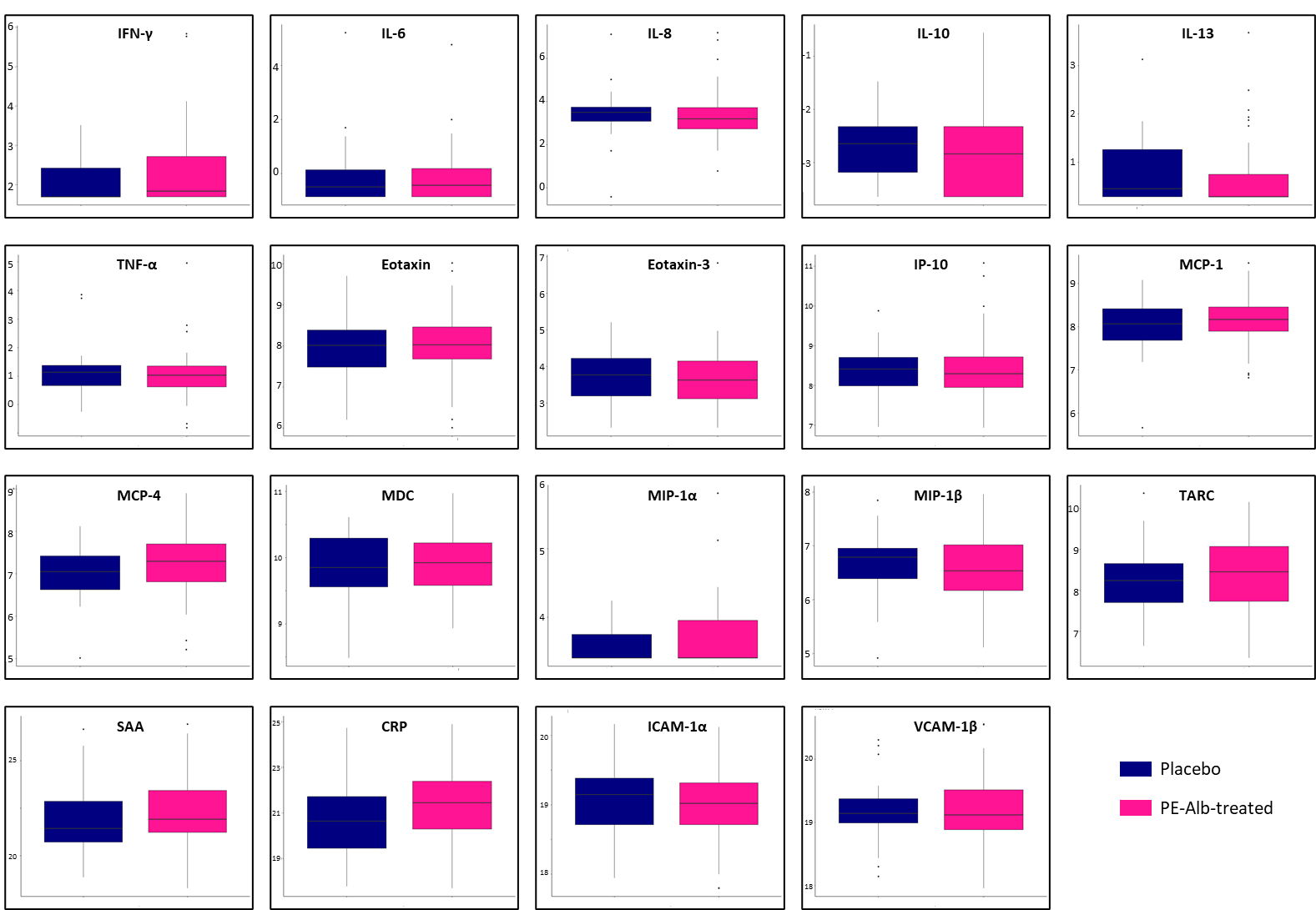


Figure S2. Comparison of inflammatory mediator levels at baseline in serum. PE-Alb: plasma exchange with albumin replacement


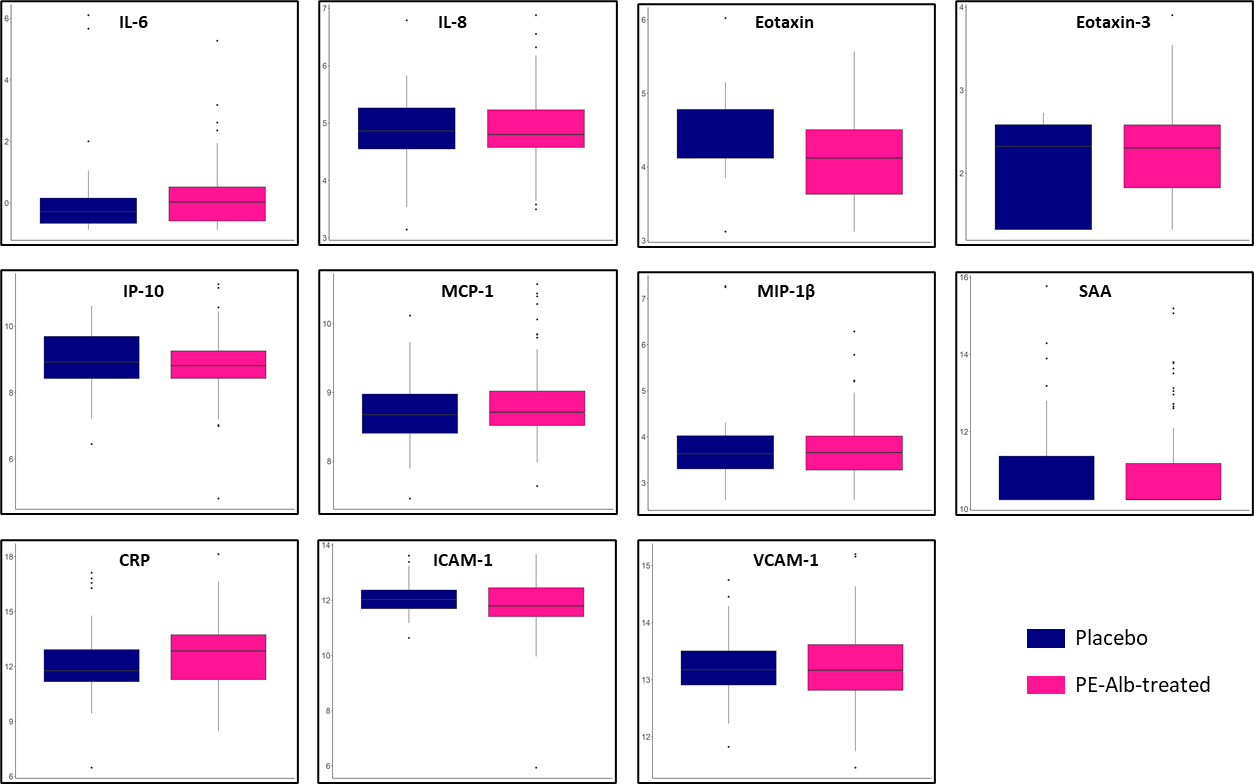


Figure S3. Comparison of inflammatory mediator levels at baseline in cerebrospinal fluid. PE-Alb: plasma exchange with albumin replacement


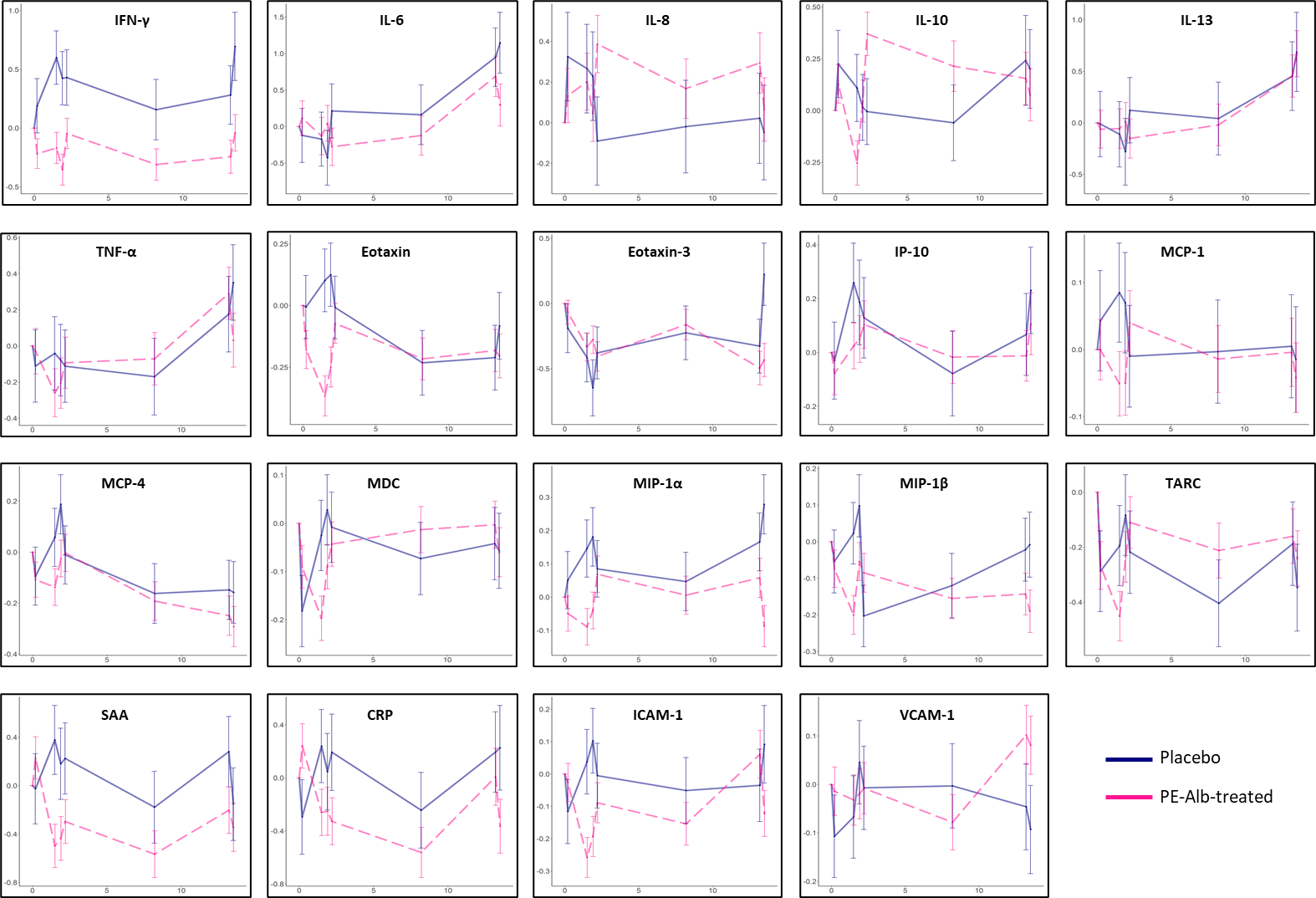


Figure S4. Lasting effect of inflammatory mediators in serum. Least square means (± standard error) was used to plot the changes over time with respect to the baseline for each mediator individually. PE-Alb: plasma exchange with albumin replacement


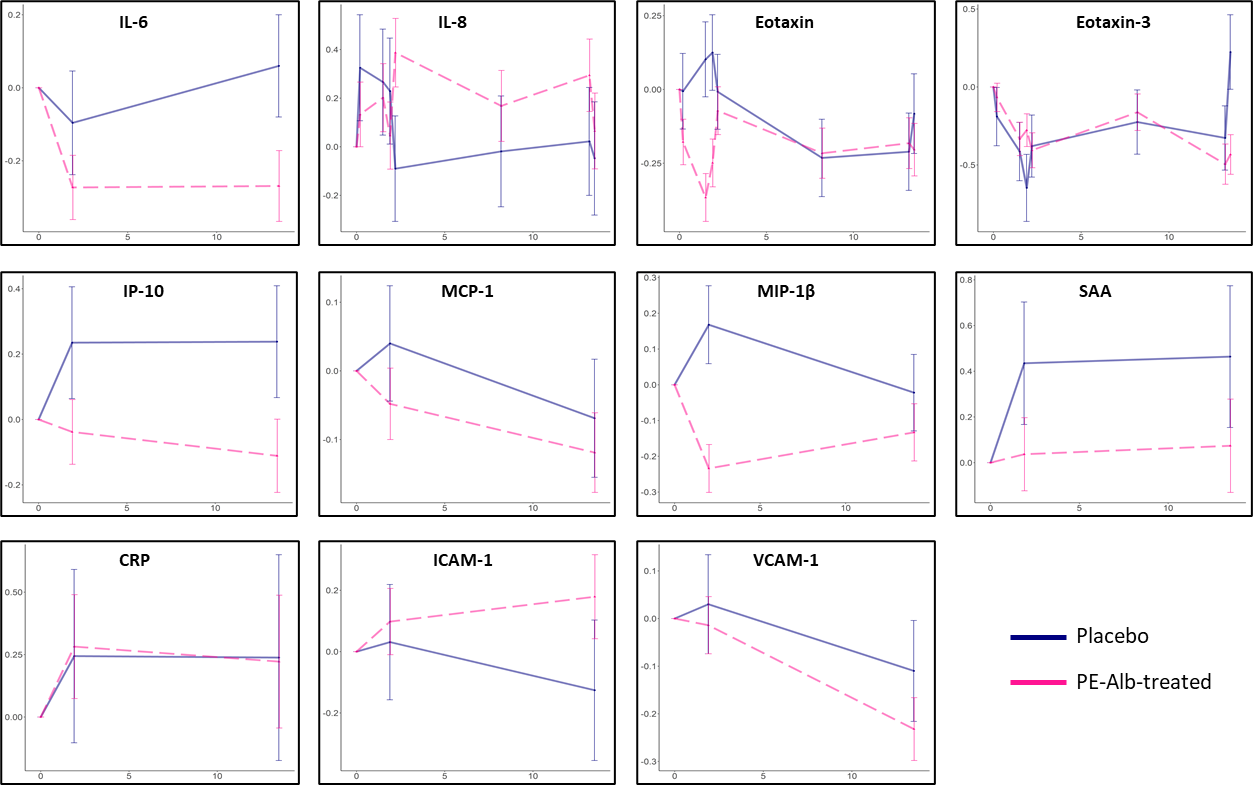


Figure S5. Lasting effect of inflammatory mediators in CSF. Least square means (± standard error) was used to plot the changes over time with respect to the baseline for each mediator individually. PE-Alb: plasma exchange with albumin replacement

Figure S6. Plot of the changes in inflammatory mediator levels (effect size of the three arms treated with plasma exchange with albumin replacement with respect to the placebo arm) before and after the LVPE 1 session (acute effects). See Figure 1 for details of plasma exchange sessions, treatment periods, visits, and treatment patient groups in the AMBAR trial

Figure S7. Forest plot of the results of inflammatory mediator analysis performed with measured variables to confirm that the longitudinal association found between the clinical outcome endpoints and the levels of MIP-1α in patients treated with plasma exchange with albumin replacement was not mediated by any other variable. Significance of the triple interaction term (Inflammatory mediator level∗Treatment_group∗Visit) was maintained regardless the hypothetical variable assessed.
